# The application and research progress of anti-angiogenesis therapy in tumor immunotherapy

**DOI:** 10.3389/fimmu.2023.1198972

**Published:** 2023-06-02

**Authors:** Jingyao Tu, Hang Liang, Chunya Li, Yongbiao Huang, Ziqi Wang, Xinyi Chen, Xianglin Yuan

**Affiliations:** ^1^ Department of Oncology, Tongji Hospital, Tongji Medical College, Huazhong University of Science and Technology, Wuhan, China; ^2^ Department of Orthopedics, Union Hospital, Tongji Medical College, Huazhong University of Science and Technology, Wuhan, China

**Keywords:** tumor immunotherapy, anti-angiogenesis therapy, vascular normalization, tumor microenvironment, immunotherapy resistance

## Abstract

Tumor immunotherapy, as the focus of scientific research and clinical tumor treatment in recent years, has received extensive attention. Due to its remarkable curative effect and fewer side effects than traditional treatments, it has significant clinical benefits for the treatment of various advanced cancers and can improve cancer patient survival in the long term. Currently, most patients cannot benefit from immunotherapy, and some patients may experience tumor recurrence and drug resistance even if they achieve remission overcome. Numerous studies have shown that the abnormal angiogenesis state of tumors can lead to immunosuppressive tumor microenvironment, which affects the efficacy of immunotherapy. Actually, to improve the efficacy of immunotherapy, the application of anti-angiogenesis drugs to normalize abnormal tumor vessel has been widely confirmed in basic and clinical research. This review not only discusses the risk factors, mechanisms, and effects of abnormal and normalized tumor angiogenesis state on the immune environment, but summarizes the latest progress of immunotherapy combined with anti-angiogenic therapy. We hope this review provides an applied reference for anti-angiogenesis drugs and synergistic immunotherapy therapy.

## 1 Introduction

Nowadays, immunotherapy is an emerging hotspot and is widely used in tumor treatment research and clinical application (1, 2). It plays a role in killing cancer cells and tumor tissues by activating autoimmune functions (3, 4). Immunotherapy has been widely reported to have excellent anti-tumor ability and prolongs patient survival, in basic research, preclinical studies, and clinical applications in multiple cancer types (5–7). However, most patients fail to or have limited benefit from this therapy (8, 9). The reason for this problem may be the presence of various mechanisms by which tumors suppress the immune system, resulting in the inability of immunotherapeutic agents to work, making the treatment much less effective, if not ineffective (10–12). Among them, the pathogenesis of tumor vasculature is one of the important triggers leading to the formation of the immunosuppressive tumor microenvironment (13). Abnormal vasculature can have a series of consequences, such as blood flow disturbance, acid accumulation, decreased blood perfusion, obstruction of drug delivery, and diffusion, leading to treatment failure, tumor growth, and metastasis (14, 15). Currently, the problem of immunotherapy resistance due to pathological tumor vasculature cannot be well resolved, and with the rapid spread of immunotherapy, this problem may become more serious in the future.

In contrast to immunotherapy, anti-angiogenesis therapies were discovered and studied much earlier (13). The role of tumor angiogenesis in the developmental process of tumors has been controversial. On the one hand, the new tumor vasculature is believed to provide nutrients and oxygen to the proliferation and distant metastasis of tumor cells, which led to the proposal of antiangiogenic therapy (16); on the other hand, it was found that the excessive elimination of tumor vasculature after antiangiogenic therapy may increase hypoxia in the tumor area and increase the difficulty of drug delivery, thus leading to the failure of patient treatment to meet expectations (17). Although the debate on tumor vasculature continues, it is now accepted that tumor vasculature can be categorized into physiological and pathological vasculature (17).

A notable distinction between the two is that the pathological vasculature can lead to the formation of an abnormal structural and functional vascular system, which is the main source of supply of nutrients required for tumor growth and metastasis, yet drugs are difficult to deliver through pathological abnormal tumor vessels (14, 15, 17). To restore the tumor vascular system, Dr. Rakesh K. Jain suggested that suitable doses of anti-angiogenesis therapy can lower tumor blood flow by improving tumor perfusion and blood flow as well as decreasing vascular permeability and interstitial fluid pressure to normalize the tumor vascular system (18). The dynamic concept of “vascular normalization” may be more applicable to the clinical application of multiple combination therapy regimens for multiple cancer types than the idea of eliminating or preserving tumor vasculature.

Anti-vascular drug-based vascular normalization therapies have attracted much attention since their introduction, but resistance may occur to receiving vascular normalization therapy alone, making it difficult to achieve significant and effective long-term outcomes (19, 20). It has been reported that the vascular normalization effect of tumors induced by different mechanisms in combination with multiple oncological therapies (radiotherapy, chemotherapy, immunotherapy) can effectively increase the efficacy and create synergistic effects (14, 21, 22). This review is focused on the synergistic effects of anti-angiogenesis drugs with immunotherapy, provides a comprehensive overview of the process of pathogenesis and normalization of tumor vasculature, the characteristics of tumor vasculature, and its effects on immunotherapy, and summarizes the latest understanding and progress of immunotherapy in combination with anti-angiogenesis therapy.

## 2 Abnormal vascular environment of tumors

Angiogenesis can usually happen under physiological conditions, including during embryonic development, wound healing, bone repair, and regeneration (23, 24). It can also occur in pathological conditions, such as tumors, immune system diseases, inflammatory responses, and hematological diseases. Pathological angiogenesis is an important part involved in disease onset and progression (15). It has a significant role in tumor progression, mainly by transporting gases and nutrients for tumor cells, assisting tumor cell dissemination, and establishing an immunosuppressive tumor microenvironment, thus accelerating tumor progression (25). The following section summarizes the process, characteristics, and inducing factors of pathological tumor angiogenesis and its effects on the tumor microenvironment.

### 2.1 Tumor angiogenesis process

The growth and metastasis of tumor tissues depend on the gas and nutrients supplied by blood vessels. When there is no pathological vascular supply of “nutrients” in the tumor microenvironment, the tumor growth is dormant, and its diameter rarely exceeds 2-3 mm. When the primary tumor lesions or the metastatic tumor lesions develop continuously, the metabolic capacity of oxygen, nutrients, and metabolites provided by blood vessels gradually becomes insufficient (26). Therefore, tumor development requires the generation or recruitment of new blood vessels through a process that promotes angiogenesis. When tumors are enriched with pathological blood vessels, tumor tissue can remove its metabolites by taking up nutrients and oxygen through additional blood perfusion, thus affecting tumor pathophysiology, growth, invasion, metastasis, and response to various treatments (27). It has been reported that pathological vascular network formation in tumor microenvironment is mainly constructed in five ways: (1) angiogenesis, which refers to the new blood vessels formation based on the original microvascular network of the body by budding induced by various factors; (2) vasculogenesis, in which tumors form new blood vessels by recruiting endothelial cells of blood and bone marrow origin; (3) vascular intussusception, in which tumor tissues form a tumor vascular network by invading the surrounding mesenchyme; (4) mosaic vessels, in which tumor cells themselves form abnormal blood vessels by acting together with endothelial cells; (5) vascular mimicry, in which tumor cells mimic and replace endothelial cells to form lumen-like structures and thus function. In these ways, the tumor acquires a new blood supply, thus providing sufficient “nutrients” for its progression (14, 15, 25).

### 2.2 Tumor pathological vascular characteristics and pathology

**Figure 1:**
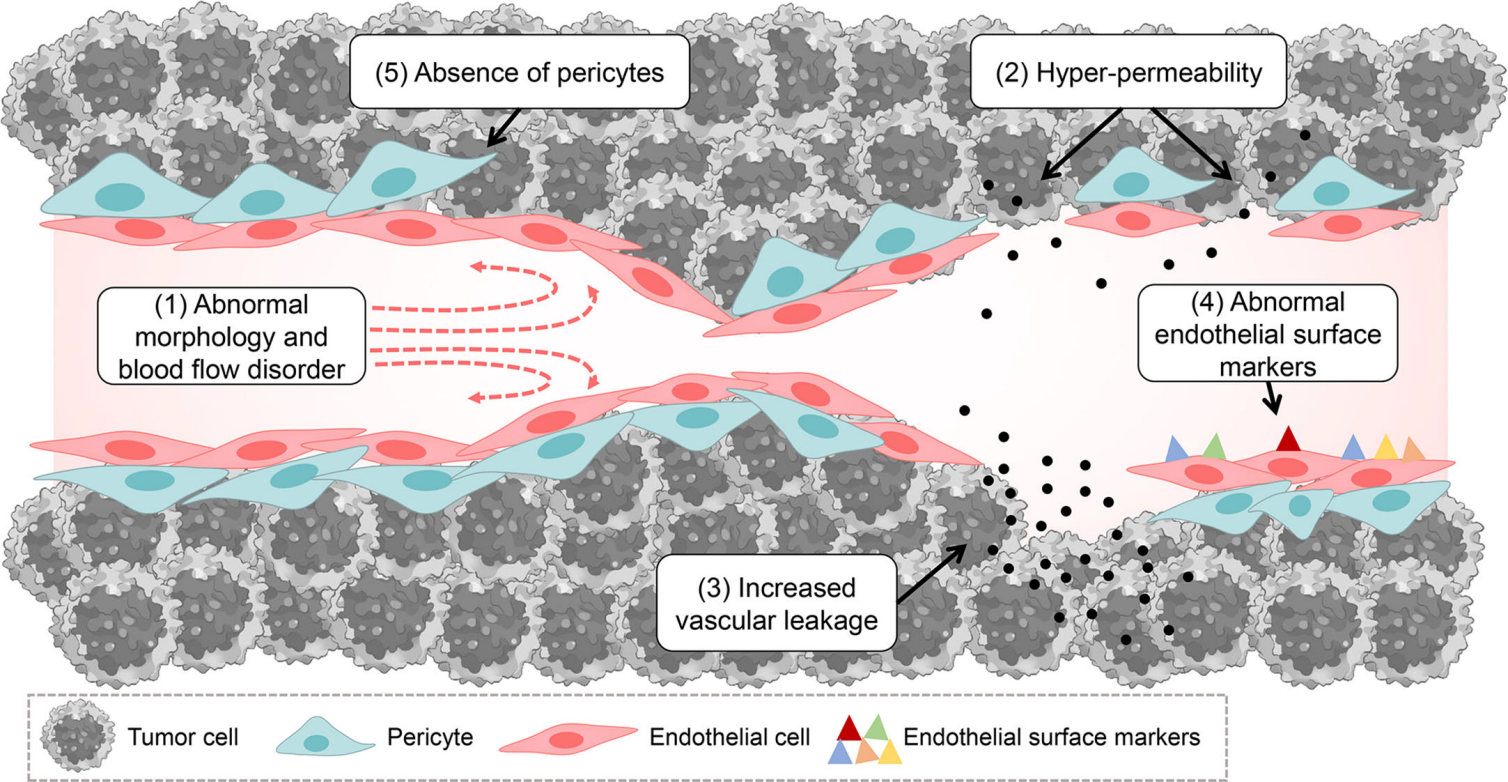
Tumor pathological vascular characteristics and pathology.

Tumor pathological vessels have many abnormal pathological characteristics compared with normal functional vessels, which may be important conditions to promote tumor progression and build abnormal tumor microenvironment. These include the following: (1) abnormal tumor vessel morphology and blood flow disorder: compared with normal vessels, tumor vessels have dysfunctional perfusion, disordered arrangement, tortuous vessel morphology, abnormal expansion and excessive branching shunts (14, 28); (2) hyper-permeability: tumor abnormal vessels have more abnormal pores (endothelial pores, vesicles and transcellular pores) in the vessel wall compared to normal vessels, and their interendothelial junctions are widened, basement membrane is discontinuous or absent, and the endothelial cells are abnormally shaped, overlapping each other and can protrude into the lumen (14, 15, 29, 30); (3) increased vascular leakage: the above-mentioned reasons can lead to increased vascular leakage, which disrupts the metabolic balance between tumor vascular leakage and normal lymphatic return, thus increasing the hydrostatic pressure within the tumor tissue (29); (4) abnormal endothelial surface markers: tumor vascular endothelial cells may express fewer surface markers, such as adhesion factors of cells, which may be one of the reasons why it is difficult for immune cells to accumulate in the tumor area through the blood vessels (15, 31); (5) absence of pericytes: compared to normal functional vessels, tumor abnormal vessels may lack pericytes (32), which mainly function to keep the vessel in a “quiescent” state, where they provide the necessary vasoactive control to adapt to metabolic demands and are a necessary barrier to protect the vessel from changes in oxygen or hormonal homeostasis (33). The above pathological features of tumor abnormal blood vessels eventually lead to a series of consequences, such as tumor growth, metastasis, accumulation of acidic metabolites, impairment of effective blood perfusion, impairment of immune cell infiltration, obstruction of drug delivery and diffusion, etc., thus degrading the efficacy of tumor therapy and accelerating tumor progression (**Figure 1**).

### 2.3 Factors inducing pathological angiogenesis in tumors

**Figure 2:**
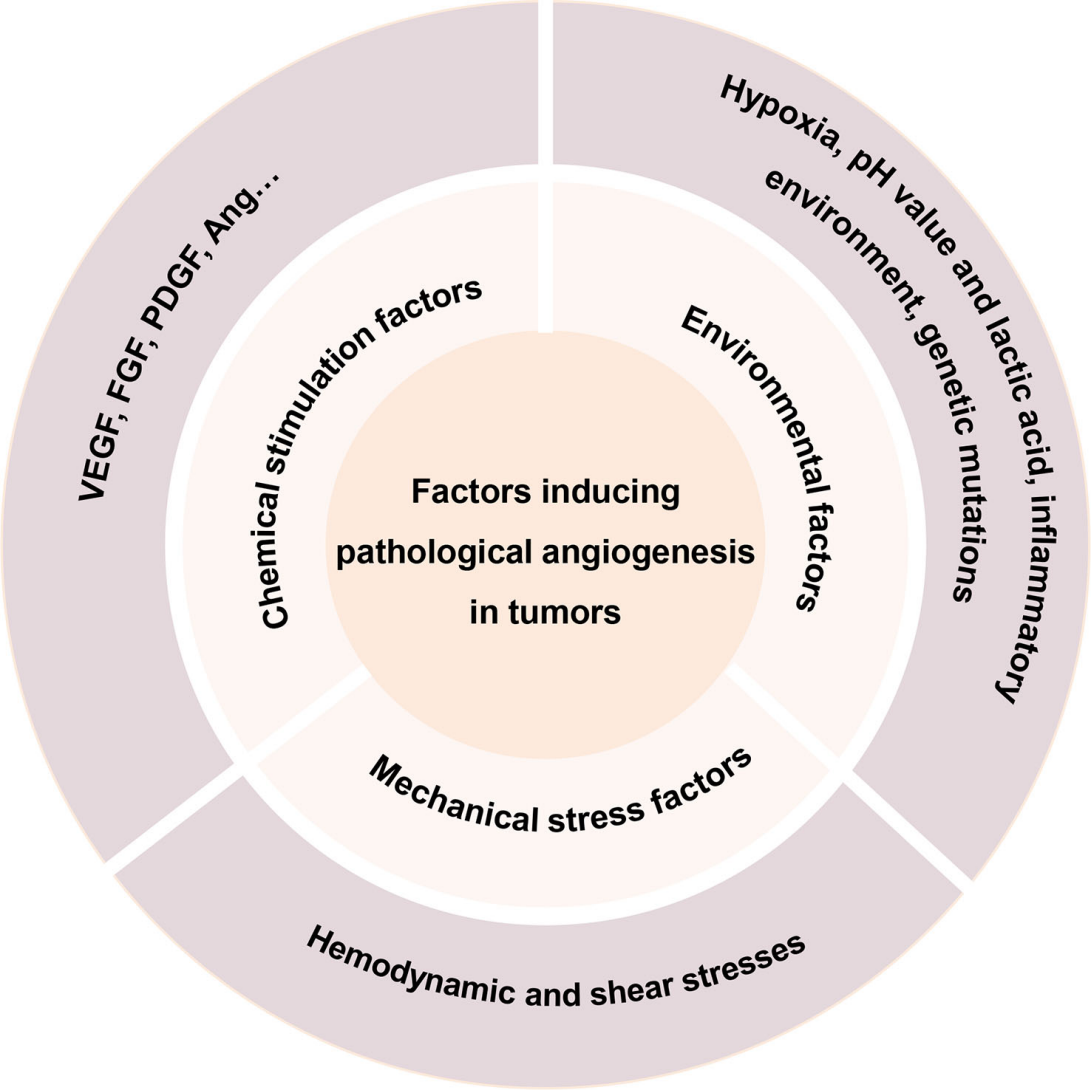
Factors inducing pathological angiogenesis in tumors.

Pathological angiogenesis is among the hallmarks and key factors related to the development of cancer. The complex interactions between these molecular factors and their effects on vascular structure and function in different environments have been gradually discovered and elucidated (15, 34). This section summarizes the various factors that have been proposed to promote angiogenesis in recent years (**Figure 2**).

#### 2.3.1 Environmental factors

##### 2.3.1.1 Hypoxia

Hypoxia stimulates angiogenesis in a variety of diseases (15). When tumors grow too far from vessels that supply blood, tumor tissues become hypoxic. Several studies have shown that tumor tissue hypoxia activates mainly Hypoxia-Inducible Factor-1 (HIF-1), which induces the expression of numerous angiogenic factors, such as Nitric Oxide Synthase (NOS), Vascular Endothelial Growth Factor (VEGF), Platelet-Derived Growth Factor (PDGF), Angiopoietin-2 (Ang 2), and others, promoting pathological angiogenesis (35, 36). In addition, VEGF is associated with a Hypoxia Response Element (HRE), which can also be activated in the presence of reduced oxygen supply, thus stimulating angiogenesis in hypoxic tissues to restore oxygen and nutrient supply to hypoxic areas (37).

In addition to directly activating relevant pathways or responses, hypoxia can promote angiogenesis by affecting the energy metabolic processes of tumor cells. Although many cancer cells are inherently energized by aerobic glycolysis (characterized by the Warburg effect), while in a hypoxic state they upregulate a series of genes that promote glycolysis, including glucose transporter protein 1/3, hexokinase, 6-phosphofructokinase, aldolase A/C, glyceraldehyde-3-phosphate dehydrogenase, and pyruvate kinase, among others (38). The activation of these enzymes contributes to the production of high levels of pyruvate, the end product of glycolysis, which can also be upregulated by the lactic acid converted from lactate dehydrogenase A (38). The role of lactate on tumor angiogenesis is described in the next subsection.

##### 2.3.1.2 pH value and lactic acid

In tumor tissues, an abnormal acidic pH environment may result from a variety of factors, including hypoxia, accumulation of acidic metabolites, and so on. Lactate, as the main product of tumor metabolism, is mainly derived from the above-mentioned glycolytic process and glutamine catabolic process (39, 40). HIF-dependent and HIF-nondependent pathways, both of which involve include Monocarboxylate Transporter 1 (MCT1)-mediated lactate import and subsequent inhibition of Prolyl Hydroxylase (PHD), increase tumor-derived lactate-induced endothelial cell activation and angiogenesis (41, 42). Tumor-derived lactate in the HIF non-dependent pathway is taken up via MCT1 by the surrounding endothelial cells, inducing angiogenesis in a ROS and NF-κB/IL-8-dependent manner (42). In the HIF-dependent pathway, however, MCT1-mediated lactate accumulation in endothelial cells inactivates PHD and stabilizes HIF-1α, which then induces transcriptional expression of a series of pro-angiogenic factors to promote angiogenesis (41). In addition, lactate has been reported to directly regulate tyrosine kinase receptors in some vascular endothelial cells, including VEGFR2, and also protect the N-Myc downstream regulatory gene 3 (NDRG3) protein from the hypoxic environment, thereby promoting angiogenesis during hypoxia (39). During tumor cell metabolism, MCT1/4-mediated lactate efflux is accompanied by symport efflux of H+, resulting in an acidified extracellular environment. In addition to ensuring that the tumor cells are at a pH level suitable for rapid growth, the resulting acidic tumor microenvironment can generate an inwards directed proton gradient across the plasma membrane of cancer cells, providing driving force for the proton-coupled in the cancer cell, and to enhance the availability of selective nutrients (39).

##### 2.3.1.3 Inflammatory environment

Angiogenesis and inflammatory exudation are hallmarks of inflammatory diseases in many organs. Angiogenesis and inflammation usually share common pathways, stimuli, etc., and both biological processes are also closely associated with cancer (43).

Approximately 15-20% of malignancies are reported to be initiated or exacerbated by inflammation. In the inflammatory tumor environment, a series of inflammatory factors secreted by immune cells, and autocrine by tumor cells like VEGF, transforming growth factor-β (TGF-β), fibroblast growth factor (FGF), PDGF, Tumor Necrosis Factor (TNF), and Interleukin (IL), among others, can stimulate angiogenesis in the tumor region (44, 45). In addition, in the tumor microenvironment, activation of some inflammation-related pathways (NF-kB, Nrf2, etc.) and some activated cellular components (macrophages, vascular endothelial cells) can also stimulate tumor angiogenesis directly or indirectly (46).

##### 2.3.1.4 Genetic mutations

Tumor gene mutations may also be involved in the angiogenesis process. VEGF production may be affected by the loss of tumor suppressor genes or increased expression of tumor-promoting genes, such as mutations in K-ras and H-ras can promote upregulation of VEGF expression (47). VEGF levels have also been reported to increase because of mutations in several oncogenes, including v-Src, HER-2, and EGFR, which activate a series of pathways (48). Moreover, PI3K/Akt signaling pathway activation has been associated with VEGF production by inhibiting PI3K signaling leading to decreased expression of VEGF and Matrix Metalloproteinase (MMP), which affects tumor development (49). Thus, dysregulation of multiple oncogenes jointly involves in regulating tumor angiogenesis.

#### 2.3.2 Mechanical stress factors

Hemodynamics and shear stresses in the tumor vasculature are also important factors affecting angiogenesis. First, the abnormal structure of the tumor microvascular system increases the geometric and viscous resistance to blood flow, leading to flow disturbances and elevated interstitial fluid pressure, which results in under-perfusion of the tumor region despite high shear stress on the vessel wall (50). In most cases, vascular endothelial cells are mitotically quiescent under healthy conditions (51), and are constantly subjected to shear stresses during circulation, including tension from extracellular matrix strain, and pressure in the blood vessels (Blood pressure), among others. In normal physiological settings, the shear stress level experienced by vascular cells can vary widely; for example, in arteries the shear stress range is 1-7 Pa, while in veins it is 0.1-0.6 Pa (50, 52). Following an increase in shear stress sensed by the vascular endothelium, the vascular endothelium is activated into an angiogenic state, which may be mediated by NOS-NO (53, 54). In addition, changes in shear stress can affect the process of angiogenesis by influencing gene expression in vascular endothelial cells, such as VEGF-A, Neuropilin-2, and MMP1/14, among others.

#### 2.3.3 Chemical stimulation factors

##### 2.3.3.1 Vascular endothelial growth factor

VEGF regulates a variety of angiogenic responses by binding to certain membrane-bound, soluble vascular endothelial growth factor receptors (VEGFR) or their coreceptors (44). The VEGF family includes VEGF-A, VEGF-B, VEGF-C, VEGF-D, and placental growth factor (PlGF1-4) (55). VEGF binds to three VEGFRs that are structurally related, which include VEGFR1, VEGFR2, and VEGFR3. These comprise three structural domains: a tyrosine kinase intracellular domain, a transmembrane, and an Ig-like extracellular ligand-binding region (56). VEGFR1, VEGFR2, and VEGFR3 are mainly expressed in vascular smooth muscle cells, vascular endothelial cells, and lymphatic endothelial cells, respectively (56, 57). The biological functions of VEGF after binding to receptors involve tumor angiogenesis (VEGF-A, PlGF), new blood vessels maintenance (VEGF-B), lymphangiogenesis and angiogenesis (VEGF-C), generation (VEGF-C/D), vascular permeability (VEGF-A/C), chemotaxis (VEGF-A, PlGF), migration (VEGF-A, PlGF), differentiation (VEGF-D), and survival (VEGF-A/B/C, PlGF) (17).

##### 2.3.3.2 Fibroblast growth factor

FGF has been shown to stimulate differentiation, proliferation, migration, morphogenesis, and survival in the endothelial cells, as well as extracellular matrix degradation, by inducing the secretion of proteases (including fibrinogen activator and metalloproteinases) and vascular maturation (58, 59). The FGF family’s angiogenic effects are dependent mainly on the activity of FGF-1 and FGF-2, which play an important role in wound healing, by inducing the proliferation of fibroblasts and endothelial cells, which are required for the production of granulation tissue needed for the healing process (58, 60). FGF-1 (acidic FGF) is the most broadly acting protein in its family. It plays a role in angiogenesis (stimulates the differentiation and proliferation of vascular-associated cell types) and binds to seven fibroblast growth factor receptor (FGFR) subtypes. FGF-2 (basic FGF) is an essential pleiotropic regulator of differentiation, proliferation, and migration of vascular endothelial cells, and vascular-specific cell survival. Inhibition of the FGF-2 signaling pathway, for example, leads to impaired vascular endothelial cell junctions and increased vascular permeability (44, 58).

##### 2.3.3.3 Platelet-derived growth factor

PDGF-A, PDGF-B, PDGF-C, and PDGF-D are four different polypeptide chains in the PDGF family that are inactive in their monomeric forms. After protein translation, PDGFs bind to monomeric chains through amino acid disulfide bonds and transform into physiologically active forms. Four homodimers (PDGF-AA/BB/CC/DD), and one heterodimer (PDGF-AB) of PGDF have been identified (61, 62). Dimeric PDGF heterodimers can specifically bind to a couple of PDGF receptor tyrosine kinases (PDGFR-α/β) producing certain cellular effects. The involvement of PDGFs in angiogenesis has been reported to include the promotion of vascular development and generation, by causing the proliferation and survival of vessel wall cells (62, 63).

##### 2.3.3.4 Angiopoietin

Ang is a protein family that includes Ang1, Ang2, Ang3, and Ang4, sharing a common amino-terminal coiled-coil domain and a carboxyl terminal fibrinogen-like domain (64). Angiogenesis and vascular repair are linked to Ang, with Ang1 and Ang2 being the main subtypes engaged in vascular homeostasis regulation (65). Ang1 is expressed in the endothelial circumference oxyntic cell (e.g., smooth muscle cells) and pericytes, fibroblasts, other non-vascular stromal cells, and tumor cells. As a major Tie2 agonist ligand, it exerts biological effects mainly in combination with the tyrosine kinase Tie2. Ang1 plays a vital role in regulating vascular maturation, stability, and remodeling, and in maintaining the stability of the entire normal vascular system during embryonic development via promoting endothelial cell survival, non-permeability, and endothelial-mural cell interactions. Ang2, on the other hand, is produced by stimulation of hypoxia, VEGF, and shear stress and plays a role opposite to that of Ang1, promoting vascular wall instability by Tie2 competitive inhibition and activation of integrin. Additionally, Ang2 is associated with stimulation of vascular permeability and degeneration, pericyte detachment, and lymphangiogenesis (64–66).

##### 2.3.3.5 Other factors

In addition to the factors mentioned above that affect tumor vasculature, there are a series of factors involved in the regulation of angiogenesis such as HIF, Hepatocyte Growth Factor (HGF), TNF, and TGF-β (60, 67). They all function in positive or negative angiogenesis after binding to the corresponding receptors.

### 2.4 Effects of abnormal tumor vasculature on immunotherapy

Pathological tumor vasculature proceeds in a dysregulated manner driven by pro-angiogenic factors. Tumor vasculature is characterized by structural abnormalities such as uneven distribution, tortuosity, dilatation, and perivascular cellular insufficiency, as well as functional ischemia, hypoxia, and vascular hyperpermeability, resulting in a tumor microenvironment characterized by hypoxia, acidity, patchy hypoperfusion, and high interstitial fluid pressure. All of these factors can have a significant impact on the immunotherapeutic response and may affect the proliferation, infiltration, survival, and function of immune cells, thus transforming the tumor microenvironment into a suppressed state (13).

#### 2.4.1 Affecting infiltration of immune cells in the tumor region

The interaction of a series of immune cells is the basis of the body’s anti-tumor immune response. For infiltration into the tumor and integration into the tumor microenvironment, immune cells need to first enter the tumor vasculature, adhere to the endothelium and then transit through the vascular wall. The pathological tumor vasculature makes it more difficult for immune cells to enter the tumor area: on the one hand, the high permeability of the tumor vasculature due to multiple causes and the high interstitial fluid pressure state due to leakage make it necessary to overcome a greater pressure difference for immune cells to infiltrate from the vasculature to the tumor area (29); on the other hand, some pro-angiogenic molecules, such as VEGF, can regulate immune cells transit to tumors by affecting the expression of adhesion molecules on vascular endothelial cells and immune cells, such as the integrin ligand intercellular adhesion molecule 1 and vascular cell adhesion molecule 1 (68). These abnormal blood vessels associated factors prevent immune cells from entering the tumor tissue region from the immune organs and circulation, thereby inhibiting their anti-tumor effects.

#### 2.4.2 Promoting the formation of immunosuppressive tumor microenvironment

**Figure 3:**
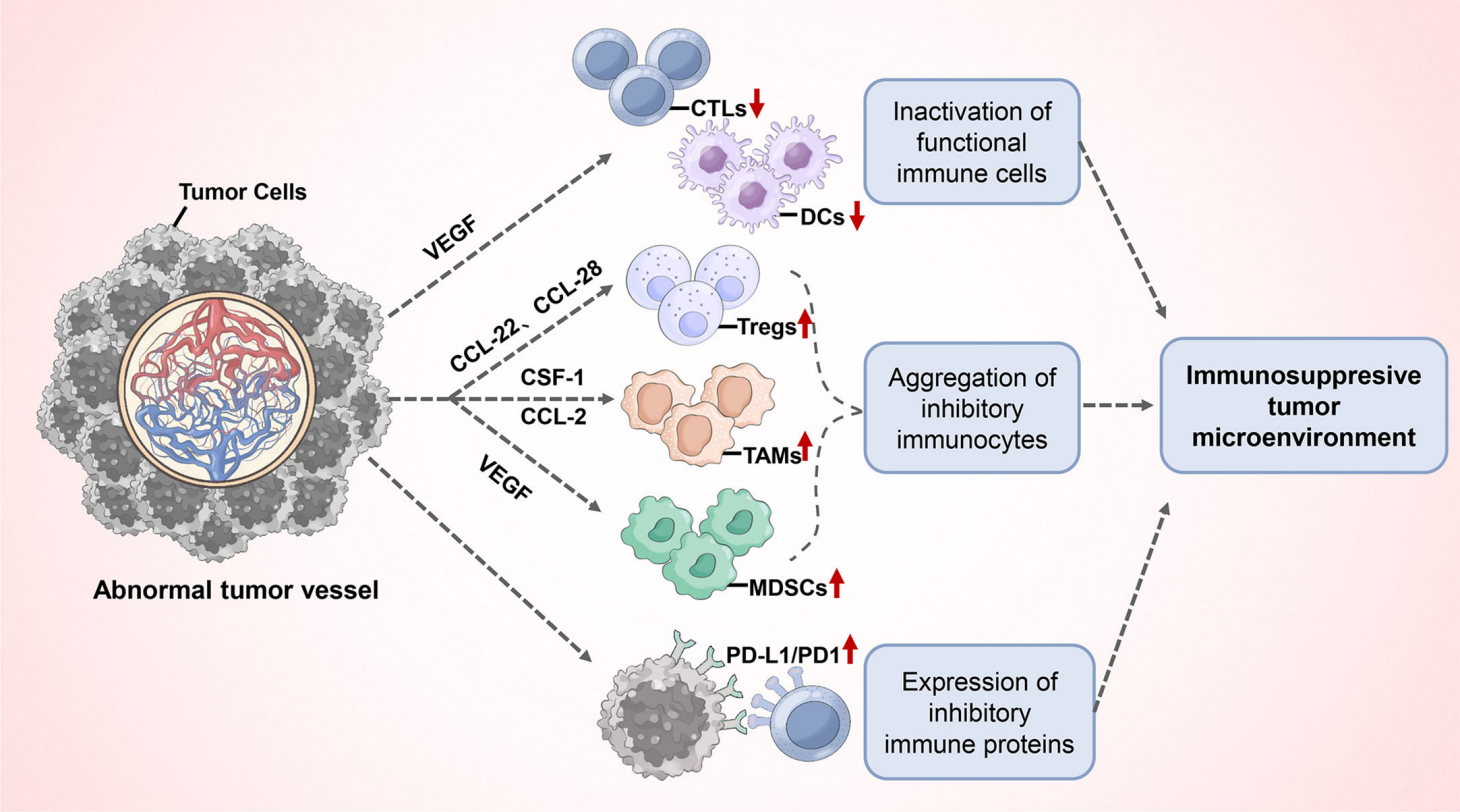
Abnormal tumor angiogenesis promotes the immunosuppressive tumor microenvironment formation.

Furthermore, abnormal tumor angiogenesis promotes the immunosuppressive tumor microenvironment formation, which consists mostly of the following (**Figure 3**): (1) inactivation of functional immune cells: the presence of VEGF in the circulation and blood vessels can affect the function and activation of immune cells. Increased VEGF directly inhibits the transport, proliferation and functions of cytotoxic T lymphocytes (CTLs) (69, 70). In addition, VEGF can inhibit the maturation and antigen presentation process of Dendritic Cells (DCs), thereby hindering T cell activation and reducing T cell-mediated anti-cancer immune responses (21, 71, 72); (2) Aggregation of inhibitory immunocytes: Although all types of immune cells can infiltrate the tumor parenchyma via functional tumor vasculature, inhibitory immune cell populations such as M2-type tumor-associated macrophages (TAMs), regulatory T cells (Tregs), and Myeloid-derived suppressor cells (MDSCs) appear to have a priority in accumulating in tumors for a variety of reasons. First, tumor hypoxia induces the upregulation of chemokine ligands CCL-22 and CCL-28, which preferentially recruit Tregs into tumors (73, 74). In addition, tumors typically express high levels of growth factors, which include colony-stimulating factor (CSF-1) and chemokine ligand (CCL-2), that attract monocytes to the parenchyma of the tumor and allow their differentiation into macrophages. Subsequently, the hypoxic environment of the tumor leads to the differentiation or polarization of tumor-infiltrating myeloid cells into M2-like TAMs with high expression of Arg1 and IL-10 (75, 76). MDSCs can accumulate in the tumor microenvironment and peripheral immune organs in response to excess VEGF and suppress T cell and DCs functions through multiple mechanisms (76); (3) Expression of inhibitory immune proteins: in response to pathological angiogenic stimuli, the expression of programmed cell death ligand 1 (PD-L1) is upregulated on M2-type TAMs, aberrant phenotypic vascular endothelial cells, and tumor cells. It binds to programmed cell death protein-1 (PD-1), expressed on T cells, thereby inhibiting their anticancer activity (77). Immunosuppressive factors like indoleamine 2,3-dioxygenase (IDO), IL-6, and IL-10 have also been shown to be upregulated (17, 78). Overall, tumor pathological angiogenesis causes functional immune cell inactivation, and the aggregated immunosuppressive cells, together with the tumor cells and endothelial cells of abnormal tumor vessels, promote angiogenesis and the formation of a local or systemic immunosuppressive environment by producing cytokines and growth factors including Ang2, VEGF, TGF-β, and IL-10.

## 3 Effects of vascular normalization on the immune environment

Since abnormal tumor angiogenesis due to the causes mentioned above leads to an immunosuppressive microenvironment in the tumor area, vascular normalization therapy may be the preferred adjuvant therapy to address the immunotherapy resistance caused by this condition.

### 3.1 Conception and characteristics of vascular normalization

Tumor abnormal vessels are usually characterized by structural abnormalities such as uneven distribution, tortuosity, dilatation, and pericyte deficiency as described above, which often cause lack of perfusion, hypoxia, and vascular hyperpermeability and leakage in the tumor area. The concept of vascular normalization refers to the use of appropriate amounts of anti-angiogenesis drugs to prune the abnormal blood vessels of the tumor, reversing many of the abnormal structures and functions in the tumor vasculature and leaving the tumor microenvironment in a benign, dynamic “window” suitable for receiving various anti-tumor therapies (18, 79). This “window” is usually limited, ranging from 7 days to several months, depending on the tumor type, timing, regimen, and dose. Excessive anti-vascular therapy can also cause increased ischemia and hypoxia in the tumor area, affecting the efficacy of tumor treatment and increasing the risk of tumor metastasis, among other adverse consequences. “Normalized” blood vessels are mainly characterized by the restoration of the structure and permeability of the tumor vessel wall and increase of pericytes and improvement of their functions, thus restoring the perfusion and transport functions of the vessels and improving the tumor microenvironment with hypoxia, acidity, high interstitial fluid pressure and other undesirable factors for the subsequent treatment of tumors (14, 21)

### 3.2 Markers of vascular normalization

When the abnormal tumor vasculature is relieved after treatment and the pathological tumor vasculature is transformed into functional perfusion vasculature and becomes the state of “vascular normalization”, the microenvironment and vasculature in the tumor area will have the following characteristics: (1) the hypoxic state in the tumor area is relieved: the partial pressure of oxygen in the tumor area increases and the proportion of hypoxic cells decreases, which can be one of the signs of pathological vascular restoration. The increase of oxygen partial pressure in the tumor area and the decrease of the proportion of hypoxic cells can be the signs of restoration of vascular function. The improvement of tumor hypoxia can usually be determined by detecting the oxygen partial pressure in the tumor area with instrumentsor by detecting the expression of hypoxia-related proteins and pathways (HIF-1α) (80–82); (2) Vascular function restoration: When using anti-vascular drugs to achieve the reversal of tumor vascular abnormalities, firstly, it can effectively improve the density, distribution, diameter, morphology, and other characteristics of blood vessels in the tumor area. Subsequently, it can restore the integrity of the tumor vascular wall, thicken the vascular basement membrane, reduce the vascular permeability and leakage, and thus reduce the interstitial pressure of tumor tissue. The morphology and distribution of vessels and functions such as vascular perfusion can usually be evaluated using imaging methods (ultrasound, MRI, and contrast) and tissue staining methods (CD31 antibody immunofluorescence staining for labeled antibodies, etc.) (83); (3) Increased pericyte coverage: An integral part of normal and mature blood vessels are pericytes which contribute to the vascular integrity and quiescence, whereas pericyte coverage in tumor vasculature is frequently insufficient. A series of effective vascular normalization therapies can effectively recruit pericytes to the transformed functional vessel wall and restore its function while reducing diseased vessels. It has been reported that this pericyte coverage is usually expressed as the pericyte/vessel staining ratio. The staining ratio of pericyte-related indicators such as Lectin/NG2/α-SMA is usually used as the numerator, while the CD31 vascular marker staining ratio is used as the denominator and is denoted as (Lectin/NG2/α-SMA)/CD31 to calculate pericyte coverage (84, 85).

### 3.3 Effect of vascular normalization on the tumor immune microenvironment

Anti-angiogenesis therapy (vascular normalization) at the proper doses can reprogram the tumor microenvironment from immunosuppressive to immune-supportive. Firstly, functionally, vascular normalization therapy improves the state of high VEGF accumulation and hypoxia in tumor tissues and increases drug delivery and efficacy. At the immune cell level, vascular normalization represents a reduction in tumor tissue interstitial pressure and restoration of adhesion molecules on the surface of vascular wall cells and immune cells, leading to easier infiltration of immune cells in tumor tissue; subsequently, improvement of the tumor microenvironment promotes activation of functional immune cells (T cells, DCs), reducing the accumulation of suppressive immune cells (Tregs, M2-type TAMs and MDSCs) and the release of a range of immunosuppressive factors (e.g. VEGF, Ang2, IL-10, and TGFβ). In addition, some inhibitory proteins and pathways in the immune microenvironment can be similarly downregulated (PD-1/PD-L1, etc.) (13, 14, 21, 44). Through this series of alterations, it was found that the improvement of immunotherapeutic efficacy by vascular normalization is mainly directed at reversing the immunosuppressive state caused by tumor vasculature.

## 4 Anti-vascular drugs combined with immunotherapy

Multiple mechanisms can lead to tumor vascular dysfunction, the most important factor being the imbalance of signaling mediated by pro and anti-angiogenesis molecules. This balance is maintained in normal tissues and general physiological processes, ensuring the normal morphology and function of the vasculature. However, in carcinogenic processes, this balance usually favors an angiogenic state and the nascent blood vessels are in an immature and functionally abnormal morphological state. Restoring this balance with reasonable doses of anti-angiogenesis agents to bring the tumor microenvironment to a “vascular normalized” state is beneficial for subsequent tumor treatment. The use of other therapies during the “vascular normalization window” has been reported to yield better results than the same agents given before or after this window (84, 86). Immunotherapy’s advent and clinical use have dramatically altered the landscape of anticancer treatment and drug research in recent years. However, often patients have not benefited from this approach. The effectiveness of immunotherapy is largely dependent on the infiltration of immune cells in the tumor microenvironment and their function. As mentioned above, normalization of blood vessels can effectively improve the tumor immune microenvironment, and therefore the combination of anti-vascular drugs with immunotherapy may be an opportunity and challenge to overcome immunotherapy resistance.

### 4.1 Main mechanism of action of anti-vascular drugs

Angiogenic factors regulate the creation of new blood vessels, including some of the more well-studied ones including VEGF, FGF, PDGF, Ang, MMP, TNF-, and HIF-1, among others. All of these angiogenic factors have the potential to be used as therapeutic targets in the treatment of tumor pathological angiogenesis.

#### 4.1.1 Growth factors

VEGF is a key factor mediating tumor angiogenesis and is overexpressed and functions in most tumors (87). Among them, the angiogenic signaling of VEGF is mainly transduced by the VEGF-A factor and its receptor VEGFR2 (88, 89). It triggers the angiogenesis signaling pathway by interacting with the extracellular structural domain of Receptor Tyrosine Kinase (RTK) found in endothelial cell membranes. VEGF and its receptor VEGFR2 are currently the main targets used in the anti-angiogenesis drug development (90). Secondly, PDGF acts on PDGFRα and PDGFRβ receptors and subsequently can promote capillary stabilization and vascular maturation processes (91, 92). In addition, FGF, especially FGF-2, promotes angiogenesis by acting on specific receptors of the endothelial cell membrane (FGFR) and inducing endothelial cells to undergo proliferation and migration (93). Ang, which is also mentioned above, is a growth factor required in the process of angiogenesis and binds mainly to a transmembrane tyrosine kinase receptor, angiopoietin receptor 2 (Tie-2), involved in the regulation of angiogenesis, vascular repair, and modulation of vascular homeostasis (94). Overall, these factors have a crucial role in angiogenesis and there is synergy between growth factor pathways to stimulate neovascularization and its stabilization.

#### 4.1.2 Cytokines

In the tumor microenvironment, tumor cells, vascular endothelial cells, and immune cells may secrete multiple cytokines to regulate many cellular signaling pathways related to the regulation of immune responses, inflammation, and angiogenesis. During the regulation of angiogenesis, multiple protein cytokines may act as activators of inflammatory and immune responses at elevated concentrations, thus causing pathological tumor angiogenesis (43, 95). Among them, IL-6 is mainly secreted by immune cells and is responsible for regulating hematopoietic, immune response, inflammatory and angiogenic processes (96, 97); IL-1 is primarily produced by bone marrow cells, and its role as a pro-inflammatory mediator has recently been demonstrated to play a key role in tumor-mediated angiogenesis, making IL-1 suppression a promising anticancer target (98); TNF-α is an inflammatory cytokine, frequently found in high levels in tumor and inflammatory tissues, produced mostly by T lymphocytes, activated macrophages, and natural killer cells, which are also involved in angiogenesis (99). In addition, many other cytokines regulate angiogenesis in the tumor microenvironment and have been considered potential targets for anti-angiogenesis therapeutic interventions in tumors.

#### 4.1.3 Transcription factors

Several transcription factors are also involved in the process of tumor angiogenesis. Among them, HIF-1 regulates the expression of genes related to cellular adaptation to hypoxic conditions (100). The hypoxia-mediated HIF-1 signaling pathway is a key factor in the regulation of tumor angiogenesis, which activates the expression of a series of angiogenic factors that regulate angiogenesis and repair (101, 102). In addition to being crucial for the angiogenesis regulation, the HIF-1 pathway also promotes the proliferation and survival of cancer cells; NF-kB is a protein complex comprised of p50, p52, p65, RelB, and c-Rel subunits as homo- or hetero-dimers, which can act as transcription factors involved in the developmental processes in tumors, such as immune and inflammatory responses. Normally, the NF-kB complex remains inactivated in the cytoplasm. IkB is phosphorylated and targeted for degradation in response to external stimulation, which activates the expression activity of NF-kB gene and triggers a cascade of downstream responses (103). NF-kB activity dysregulation, however, plays a key role in angiogenesis, inflammatory and autoimmune diseases, and cancer processes (43). The IkB kinase-β (IKK-β) is responsible for IkB phosphorylation and is part of the IKK complex. It is considered an angiogenic inducer and could serve as a potential anti-angiogenesis target (104).

#### 4.1.4 Protein kinases

The complex and diverse mechanisms of tumor angiogenesis provide many targets for clinical therapeutic interventions, and many of these protein kinases play important roles in tumor vascular regulation: (1) Cyclin-Dependent Kinase (CDK) exerts its kinase activity usually in combination with cyclins. CDK6-cyclin D1 complex, for example, has been demonstrated to have a role in tumor angiogenesis via VEGF synthesis regulation (105); (2) Protein Kinase B (Akt) is considered as a key molecule in important signaling pathways which are involved in cell growth, proliferation, and survival functions, and is a potential target for regulating abnormal tumor vasculature (106); (3) Extracellular Regulated Protein Kinases (ERK) are mainly activated by certain cytokines and growth factors, and have been shown to play key roles in several aspects of cellular functions including proliferation, differentiation, and survival. It has been noted that VEGF can induce phosphorylation of ERK, suggesting that ERK can act as a specific effector of the VEGFR signaling pathway to activate the angiogenic process (107); (4) Protein Kinase C (PKC) acts as an intracellular messenger and plays an important role in the proliferation and migration of endothelial cells. PKC is known to be involved in a variety of angiogenic signaling pathways, and inhibition of PKC has been shown to promote angiogenesis (108); (5) Casein Kinase 2 (CK2) catalyzes the phosphorylation of a variety of cytoplasmic and nuclear proteins. It is involved in the regulation of a variety of biological processes, including survival, proliferation, and cell differentiation, and there is increasing evidence that it plays a role in the regulation of angiogenesis (109).

#### 4.1.5 Other factors

In addition to the above factors often seen in tumors, many other mechanisms have been reported to stimulate tumor angiogenesis in recent years: (1) MMPs: are proteases involved in extracellular matrix degradation and are essential for a variety of biological events such as embryogenesis, cell proliferation, apoptosis, and angiogenesis. Several MMPs have been confirmed to be associated with cancer development, metastasis, and pathological angiogenesis as reported by several studies (110); (2) Cyclooxygenase-2 (COX-2): a key enzyme in the process of prostaglandin in biosynthesis, induces the onset and development of inflammation and plays an important role in many inflammatory diseases. A study showed that downregulation of COX-2 in the presence of anti-angiogenesis therapy led to a decrease in PGE2 and an increase in the efficiency of anti-VEGFR2 therapy (111). Blockade of COX-2 and VEGF signaling pathways may have a synergistic effect on tumor angiogenesis. (3) Nitric oxide (NO) is mainly catalyzed by the NOS family, including neuronal (nNOS), endothelial (eNOS), and inducible (iNOS) isoforms. It has been reported that NO may play an important role in promoting neovascularization (80), as well as in tumor angiogenesis through the activation of the Akt/eNOS signaling pathway (112). In addition, many other factors involved in the regulation of angiogenesis such as platelet-responsive protein-1, interferon (IFN) α/β, and integrins, among others, have been reported to act as potential targets for the inhibition of angiogenesis.

### 4.2 Combined application of common anti-vascular drugs and immunotherapy

As mentioned above, pathological tumor angiogenesis can cause various immunosuppressive states, which eventually lead to tumor immune escape and immunotherapy resistance, etc. In addition, the application of Immune Checkpoint Inhibitors (ICIs) in the clinic sometimes leads to serious immune-related adverse events (113), and these toxicities and side effects can usually be resolved by discontinuing ICIs therapy or reducing the dose of ICIs used (114).

Considering the aforementioned, vascular normalization therapy can effectively improve the immunosuppressive microenvironment of tumors and the delivery of therapeutic agents to tumors. The proposed strategy of combining ICIs therapy with vascular normalization may achieve better efficacy with only lower doses of ICIs, while also reducing the risk of toxicity. Based on the increasing clarity of this combination mechanism, increasing studies of anti-vascular drugs in combination with ICIs are being conducted in basic research and clinical trials. The following is a brief list of several antivascular agents that have recently received attention for use with immunotherapy in basic or clinical studies.

#### 4.2.1 Nintedanib

Nintedanib (trade name Ofev) is a potent triple tyrosine kinase inhibitor that acts primarily on VEGFR1/2/3, FGFR1/2/3, and PDGFRα/β. Nintedanib is currently approved for the treatment of idiopathic pulmonary fibrosis, locally advanced or metastatic or locally recurrent non-small cell lung cancer, systemic sclerosis, and its associated interstitial lung disease. In basic research, nintedanib in combination with anti-PD-L1 was found to be effective in enhancing the efficacy of immunotherapy, inhibiting tumor growth and metastasis, and reducing pulmonary complications in transplantation tumor models (115). In addition, it has also been reported that the application of nintedanib increased the infiltration of immune cells in the tumor area and inhibited Cancer-Associated Fibroblast (CAF), thereby increasing the antitumor efficacy of anti-PD-1 therapy (116). In terms of clinical trials, there are few studies of nintedanib combined with immunotherapy, but some patients with tumor progression after prior treatment with ICIs have been reported to be responsive to nintedanib combined with immune strategies (117).

#### 4.2.2 Bevacizumab

Bevacizumab (trade name: Avastin), which inhibits VEGF, is used to treat various types of metastatic cancers. In one study, bevacizumab combined with Atezolizumab was found to promote normalization of tumor vasculature, increase T-cell infiltration in tumor areas, and improve patient survival (118). In addition, in several clinical studies, bevacizumab combined with immunotherapy was found to demonstrate improved patient PFS and OS after combination therapy compared to monotherapy in the treatment of non-small cell lung cancer, hepatocellular carcinoma, and renal cell carcinoma (119). As an anti-angiogenesis agent targeting VEGF, bevacizumab in combination with ICIs has been demonstrated to increase immune efficacy in a variety of cancer types.

#### 4.2.3 Lenvatinib

Lenvatinib (trade name: Lenvima) is a multi-target kinase inhibitor with major targets including VEGFR1/2/3, FGFR1, PDGFR, cKit, Ret, and others. It is currently approved by the FDA for the treatment of hepatocellular carcinoma, thyroid cancer, and advanced renal cell carcinoma. In a bioinformatic analysis report on the combination of levatinib and ICIs in oncology, it was found that many studies have now suggested that the synergistic antitumor effects of this combination strategy are achieved mainly by promoting normalization of tumor vasculature, increasing immune cell infiltration, and reversing suppression of the body’s immune function (120); in addition to its anti-vascular activity, it was reported in a basic study that its combination with immunotherapy reduced the number of TAMs, increased the number of T cells and activated the IFN signaling pathway, thus increasing the immunotherapeutic efficacy (121). Moreover, in clinical trials, levatinib in combination with Pembrolizumab has been reported to show effective antitumor activity in patients with advanced hepatocellular carcinoma, renal cell carcinoma, and advanced endometrial cancer (122–124).

#### 4.2.4 Sunitinib

Sunitinib (trade name: Sutent) is a multi-target RTK inhibitor, which exerts anti-cancer effects on targets including PDGFR, VEGFR1/2/3, FLT-3, CSF-1R, Kit, and Ret. Currently, it is mainly used in the treatment of gastrointestinal mesenchymal tumors, renal cell carcinoma, and other tumors. Some studies have reported that sunitinib can improve the antitumor effect of anti-DR5 receptor immunotherapy by normalizing tumor vasculature, improving lymphatic system function, and increasing CD8+ T cell infiltration (125); some recent studies have indicated that sunitinib can reduce PD-L1 expression by regulating P62, thus improving CTL activity and immunotherapeutic efficacy (126). Furthermore, some studies have pointed out that the effects of sunitinib may be limited by MDSCs (127). In addition to its common clinical indications, sunitinib in combination with Nivolumab has been reported to be effective in improving survival in patients with advanced soft tissue sarcoma in recent clinical studies (128).

#### 4.2.5 Other anti-vascular drugs

In addition to the common anti-angiogenesis targeting drugs mentioned above, there are many similar drugs such as apatinib, sorafenib, regorafenib, thalidomide, and so on. Although their targets and mechanisms of action may be slightly different, their combination strategies with immunotherapy have shown promising efficacy in both basic and clinical studies. The results of these studies can show that vascular normalization therapy can effectively improve the efficacy of immunotherapy and provide new options and theoretical basis for clinical treatment options for immunotherapy-tolerant patients.

### 4.3 Adverse events associated with combination therapy and management

A major challenge of antiangiogenic therapy is that only transient vascular normalization is observed, and the window of normalization may be short (days to weeks) and dependent on the tumor and the dose of antiangiogenic drugs used. High doses or prolonged anti-angiogenesis therapy may lead to lower levels of tumor blood perfusion and exacerbate hypoxia, which may promote tumor development, metastasis, or compromise the efficacy of the combination of ICIs. In addition, the most common side effect of ICIs application is to cause immune-related adverse events, where disturbed immune homeostasis can lead to immune-related injury in normal tissues such as the respiratory system, skin, gastrointestinal tract, and liver system (113). It has been reported that normalization of tumor vasculature can facilitate the elimination of the immunosuppressive tumor microenvironment and improve drug delivery to the tumor, which can reduce the dose of ICIs used, thereby increasing efficacy and reducing toxicity. However, each of these two therapies has complex biological effects and may also increase the risk of toxicity if combined inappropriately. Therefore, there is an urgent need to optimize the dose, duration, and dosing sequence of anti-angiogenesis agents before combining them with ICIs to prolong patient survival.

## 5 Summary and outlook

The abnormal angiogenic state of tumors is caused by a variety of factors and is a necessary condition for tumorigenesis, development, and metastasis. The initial discovery and application of anti-angiogenesis therapy aimed to exert anti-tumor effects by blocking the formation of new blood vessels in tumors. Subsequently, it was discovered through continuous exploration that anti-angiogenesis could normalize blood vessels in tumors, which provided the basis for its combination with various anti-tumor therapies. Currently, both vascular normalization therapy and immunotherapy have become important strategies for tumor treatment, but their single applications face several bottlenecks and challenges. Although immunotherapy has shown significant efficacy in both basic research and clinical applications, it still suffers from several problems such as immune escape, tolerance, and side effects.

A large number of studies have revealed complex regulatory mechanisms between aberrant angiogenesis in the tumor microenvironment and the immunosuppressive tumor microenvironment, and have validated the therapeutic efficacy of combined immunotherapy and anti-angiogenesis therapies in a variety of tumor animal models. In addition, an increasing number of clinical trials have also validated the role and effectiveness of this combination strategy. The combination of vascular normalization therapy and immunotherapy can be considered to be effective in improving the efficacy of immunotherapy, reducing side effects, and prolonging the survival cycle of patients. However, there are still some problems with this combination strategy: the problem of maintaining a short “vascular normalization window” and the lack of markers and criteria to represent and predict vascular normalization. Moreover, further experiments and data are still needed to answer the question of resistance and side effects of anti-vascular drugs combined with immunotherapy. In conclusion, a comprehensive study in recent years suggests that vascular normalization therapy can be one of the combination options to improve the efficacy of immunotherapy. However, this treatment strategy still faces certain tests and challenges.

## Author contributions

Conceptualization, XY and JT; methodology, JT and HL; validation, ZW and YH; investigation, JT and XC; writing—original draft preparation, JT and HL; writing—review and editing, XC and CL; supervision, XY; funding acquisition, XY. All authors contributed to the article and approved the submitted version.
